# GANMasker: A Two-Stage Generative Adversarial Network for High-Quality Face Mask Removal

**DOI:** 10.3390/s23167094

**Published:** 2023-08-10

**Authors:** Mohamed Mahmoud, Hyun-Soo Kang

**Affiliations:** 1 Department of Information and Communication Engineering, School of Electrical and Computer Engineering, Chungbuk National University, Cheongju-si 28644, Republic of Korea; mohamedabokhalil@aun.edu.eg; 2 Information Technology Department, Faculty of Computers and Information, Assiut University, Assiut 71515, Egypt

**Keywords:** face mask removal, image inpainting, generative adversarial networks (GANs), attention mechanism, face unmasking, autoencoder, COVID-19, CelebA dataset

## Abstract

Deep-learning-based image inpainting methods have made remarkable advancements, particularly in object removal tasks. The removal of face masks has gained significant attention, especially in the wake of the COVID-19 pandemic, and while numerous methods have successfully addressed the removal of small objects, removing large and complex masks from faces remains demanding. This paper presents a novel two-stage network for unmasking faces considering the intricate facial features typically concealed by masks, such as noses, mouths, and chins. Additionally, the scarcity of paired datasets comprising masked and unmasked face images poses an additional challenge. In the first stage of our proposed model, we employ an autoencoder-based network for binary segmentation of the face mask. Subsequently, in the second stage, we introduce a generative adversarial network (GAN)-based network enhanced with attention and Masked–Unmasked Region Fusion (MURF) mechanisms to focus on the masked region. Our network generates realistic and accurate unmasked faces that resemble the original faces. We train our model on paired unmasked and masked face images sourced from CelebA, a large public dataset, and evaluate its performance on multi-scale masked faces. The experimental results illustrate that the proposed method surpasses the current state-of-the-art techniques in both qualitative and quantitative metrics. It achieves a Peak Signal-to-Noise Ratio (PSNR) improvement of 4.18 dB over the second-best method, with the PSNR reaching 30.96. Additionally, it exhibits a 1% increase in the Structural Similarity Index Measure (SSIM), achieving a value of 0.95.

## 1. Introduction

Image inpainting is a fascinating research area in computer vision that involves filling in missing or damaged regions of images. It has many applications, including the removal of unwanted objects, restoration of old images, style transfer between images, and generating new images. In the wake of the COVID-19 pandemic, the widespread adoption of face masks has sparked a growing interest in face inpainting, particularly in the context of face mask removal. The ultimate objective of image inpainting is to generate realistic and coherent replacements for missing or damaged regions by leveraging the surrounding information. Various traditional approaches such as interpolation [1,2], patch-based methods [3,4,5,6,7], and diffusion-based methods [8,9,10] have been used. However, recent advances in deep learning algorithms, together with the availability of large-scale datasets, have opened the way for deep-learning-based approaches [11,12,13,14,15,16,17,18,19] that produce high-quality inpainted results. Deep-learning-based methods not only excel in the field of image inpainting but also deliver remarkable outcomes across various domains, including object detection [20,21], semantic image synthesis [22], Software Project Time Estimation [23], and security applications [24].

Existing techniques for image inpainting encompass methods like interpolation and patch-based, diffusion-based, and deep-learning-based approaches. Interpolation methods estimate missing or corrupted pixels by employing mathematical models based on neighboring pixels, such as nearest neighbor interpolation, bilinear interpolation, and bicubic interpolation. Patch-based methods search for similar patches in known regions or similar images and utilize them to fill in the missing regions. By employing partial differential equations, diffusion-based methods aim to minimize the discrepancy between the original image and the inpainted image. While these traditional techniques prove effective in simple cases, they often fall short when faced with challenging inpainting tasks, particularly those involving complex and large objects. Consequently, deep-learning-based methods have emerged, especially for tasks like face unmasking.

Face unmasking presents a formidable challenge in image inpainting, as it involves the removal of face masks and the subsequent filling in of the occluded regions. Unlike other object removal tasks, face masks cover a substantial portion of the face, concealing intricate and detailed features such as the nose, mouth, and chin. Furthermore, the scarcity of large-scale datasets containing paired masked and unmasked face images for training deep-learning-based methods adds to the difficulty of generating realistic and coherent inpainted results.

**Figure 1:**
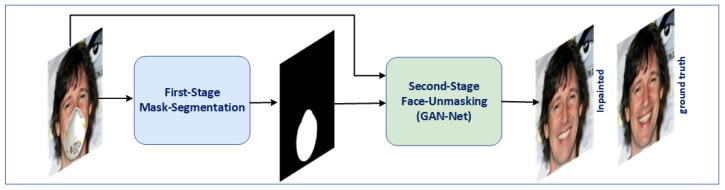
Overview of our two-stage approach for face unmasking. The first stage, Mask Segmentation, takes the masked face as input and generates a binary mask map. The second stage, Face Unmasking (GAN-Net), utilizes the masked face and the generated mask map from the first stage to generate the unmasked face.

In this work, we propose a two-stage deep-learning-based approach for face unmasking that addresses these limitations and outperforms existing methods. Figure 1 shows an overview of our approach. In the first stage, we introduce an autoencoder network modified from the U-net [25] architecture to accurately detect the mask region by generating a binary mask segmentation map. This binary mask segmentation map is a guidance mechanism for the subsequent stage. We leverage a modified autoencoder model based on the U-net architecture for binary mask segmentation. In the second stage, we employ generative adversarial networks (GANs), comprising a generator and a discriminator. Our generator incorporates a residual attention and a Masked–Unmasked Region Fusion (MURF) mechanism, while the discriminator utilizes spectral normalization to enhance training stability. Additionally, we integrated a CBAM (Convolutional Block Attention Module) block [26] as an attention mechanism in our generator network to focus on essential features and the MURF mechanism to focus only on the mask region. To train our network, we generated a synthetic dataset from the large public CelebA dataset [27], utilizing MaskTheFace [28] to address the challenge of the limited availability of paired masked and unmasked face images. We achieve qualitative and quantitative improvements compared to state-of-the-art methods through training on this dataset, even in scenarios involving large and complex masks.

Our paper introduces a novel two-stage network for face unmasking that effectively addresses the challenge of removing face masks and inpainting the occluded regions while considering the detailed facial features concealed by the mask. The key contributions of our research are as follows:

1. Proposal of a two-stage network: we present a unique two-stage approach that combines autoencoder-based binary segmentation in the first stage and a GAN-based network with residual attention and spectral normalization in the second stage for generating realistic and coherent unmasked faces.
2. Accurate binary segmentation: In the first stage, we introduce an autoencoder-based approach to achieve precise binary segmentation of the face mask. This results in a binary mask segmentation map that guides the subsequent stage.
3. GAN-based network with enhanced mechanisms: in the second stage, we leverage a GAN-based network with a residual attention mechanism in the generator and spectral normalization in the discriminator, enabling us to generate unmasked faces that closely resemble the original ones.
4. Synthetic dataset generation: to address the scarcity of paired masked and unmasked datasets, we generate a synthetic dataset using the large public CelebA dataset, enhancing the training process.
5. Superior performance: extensive experiments and evaluations demonstrate the superiority of our proposed method over state-of-the-art techniques in both qualitative and quantitative metrics, even in scenarios involving large and complex masks.
6. Real-world applications: our proposed method exhibits promising potential for real-world applications, including automated face recognition and identity verification, contributing to advancements in computer vision and image inpainting.

Our work presents a comprehensive and innovative solution for face unmasking, with practical implications for various applications in computer vision and image processing.

## 2. Related Work

In this section, we provide an overview of the existing techniques for image inpainting, with a particular focus on face unmasking. We categorize the approaches into traditional techniques, including interpolation and patch-based and diffusion-based methods, which have been widely used in the past. Additionally, we discuss the advancements made in deep learning techniques for image inpainting.

### 2.1. Traditional Techniques

#### 2.1.1. Interpolation

These techniques estimate missing or corrupted pixels based on neighboring pixels, using methods such as nearest neighbor interpolation, bilinear interpolation, and bicubic interpolation. For instance, Jassim et al. [1] introduced a statistical algorithm based on the Kriging interpolation technique, preserving spatial correlation while filling damaged regions. Alsalamah et al. [2] proposed a radial basis function (RBF) interpolation technique for image inpainting, achieving high accuracy in repairing damaged images.

#### 2.1.2. Patch-Based Methods

These methods divide the image into small patches and use similar patches from known regions to infer missing information. Criminisi et al. [3] combined texture synthesis and inpainting techniques to benefit from their advantages. Their algorithm leverages exemplar-based texture synthesis to replicate both texture and structure. They used the exemplar-based synthesis to calculate the values of the actual color. Barnes et al. [4] developed a randomized algorithm for finding approximate nearest neighbor matches between image patches in a short time, enhancing performance for interactive image editing. They provided some constraints on the synthesis process for user control. Lin Liang et al. [5] proposed a patch-sampling-based texture synthesis algorithm with high-quality textures from an input sample. Their algorithm uses the Markov Random Field (MRF) density function as a nonparametric estimation of the local conditional to sample patches to avoid mismatching features across patch boundaries. Their proposed algorithm is much faster than existing algorithms, making real-time texture synthesis possible. Simakov et al. [6] introduced a bi-directional similarity measure for summarizing visual data. They re-scaled images or videos into smaller sizes while saving as much applicable visual information as possible and minimizing the introduction of new artifacts. It has been used in various applications such as object removal, cropping, completion, and reshuffling. Soheil Darabi, Eli Shechtman, et al. [7] presented an image melding approach for synthesizing a transition region between source images without visible artifacts. They built their method on a patch-based optimization approach with three enhancements: an enriched patch search space, integration of image gradients, and a new energy function based on mixed L2/L0 norms. Their method can be used in object cloning, stitching panoramas, hole filling, and image harmonization.

#### 2.1.3. Diffusion-Based Methods

These methods use partial differential equations (PDEs) to model the diffusion process and fill in missing regions iteratively. Bertalmio et al. [8] proposed an algorithm inspired by professional restorators, allowing simultaneous filling-in of numerous regions with different structures without any limitations on the region to be inpainted. Their method is used to restore old photographs and damaged film and for the removal of text or objects and special effects. Prasath et al. [9] introduced a fast split Bregman-based implementation of total variation (TV) regularization for object removal. Biradar et al. [10] utilized the median filter as a nonlinear filter for inpainting, diffusing the median pixel from the exterior to the inner area.

### 2.2. Deep Learning Techniques

Deep learning techniques have revolutionized image inpainting in recent years by learning directly from data to fill in missing regions. Deep learning approaches have demonstrated superior performance, particularly in complex tasks like face unmasking. However, relatively few methods have been proposed for face unmasking due to the task’s complexity and the scarcity of paired masked and unmasked face datasets.

One of the earliest deep-learning-based methods for image inpainting was proposed by Pathak et al. [11], who introduced a convolutional neural network (CNN)-based method using context encoders to predict missing pixels. It achieved impressive results, but it was limited by its ability to only fill in small missing regions. Iizuka et al. [12] proposed a GAN-based network with global and local context discriminators for image completion, including objects with specific structures like faces. Yu et al. [13] presented a generative image inpainting system utilizing gated convolutions for completing images with free-form masks and guidance. Their generator has two encoders, one using contextual attention and the other using dilated gated convolution. A combination of these two encoders is used for the single decoder. Additionally, they proposed a new GAN loss called SNPatchGAN which works on patches. Their proposed model generates higher quality and more flexible results than previous methods. Nazeri et al. [14] proposed a two-stage adversarial model for image inpainting, generating edges in the first stage as a guide for completing missing regions in the second stage. The model was evaluated on several publicly available datasets and outperforms current state-of-the-art techniques quantitatively and qualitatively. Liu et al. [15] addressed artifacts in deep-learning-based methods using partial convolutions only considering valid pixels and updating masks during the forward pass.

Furthermore, we focus specifically on face inpainting, including object removal and editing faces. Li et al. [16] developed a deep generative model for face completion, generating missing pixels for key components like eyes, noses, and mouths. Their model, trained with a combination of reconstruction loss, adversarial losses, and semantic parsing loss, produces realistic and visually consistent results for large missing regions. Khan et al. [17] introduced a GAN specifically for object removal from facial images, such as removing microphones. Their two-stage approach involves an inpainter stage for coarse prediction and a refiner stage for fine detail generation. The joint loss function includes perceptual loss, reconstruction loss, and adversarial loss to ensure realistic face reconstruction. Din et al. [18] proposed a two-stage network for removing masks in masked facial images. The first stage automatically produces binary segmentation for the mask region, while the second stage synthesizes the affected region with fine details while maintaining global coherency. Their GAN-based network employs a two-discriminator setup. It first utilizes the global discriminator during training to generate the overall facial structure. Subsequently, it completes the training process using the local discriminator, which focuses on generating the masked area. Jam et al. [19] presented a novel facial image inpainting method combining Wasserstein GAN and a Reverse Masking Network (R-MNet). Their approach leverages a new loss function computed in the feature space to target only valid pixels, resulting in more realistic outputs. The method demonstrates the ability to generalize to high-resolution inpainting tasks.

Our proposed method for face mask removal surpasses traditional techniques and previous deep-learning-based approaches in terms of accuracy, handling large missing regions, realistic texture and structure replication, robustness to complex masks, and generalization to high-resolution inpainting tasks.

## 3. Approach

In this section, we present our innovative two-stage network architecture specifically designed for face unmasking. The first stage of our network focuses on binary mask segmentation using an autoencoder model. This autoencoder is crucial in generating accurate binary segmentation maps for the masked regions of the masked faces. These segmentation maps serve as a valuable guide for the second stage of our network. Moving forward, we delve into the second stage, where we elaborate on the methodology employed to complete the missing regions with fine details while ensuring the overall coherence of the facial structure. Additionally, we discuss our combined loss function, which encompasses multiple components, including reconstruction loss, adversarial loss, and perceptual loss. This comprehensive formulation of the loss function aids in producing visually plausible and realistic results. Moreover, we introduce the integration of a perceptual network, which enhances the perceptual fidelity and visual quality of the inpainted faces, further elevating the performance of our network.

### 3.1. Mask Segmentation (M-Seg) Module: Autoencoder-Based Binary Mask Segmentation

**Figure 2:**
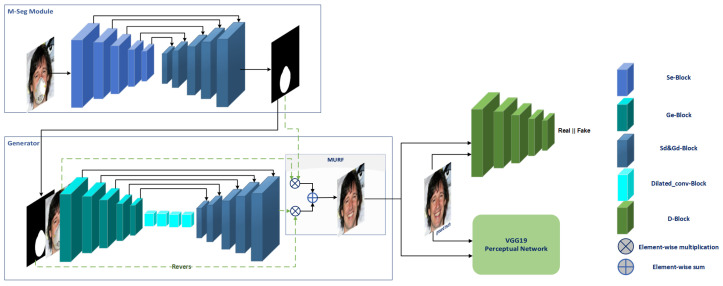
Comprehensive network architecture for face unmasking, comprising two key stages: (1) mask segmentation (M-Seg) utilizing an autoencoder architecture, and (2) face unmasking employing a GAN network. The generator module incorporates a residual attention mechanism and the MURF block, enhancing the efficacy of the unmasking process. The VGG19 network serves as the perceptual network.

In our proposed approach, the first stage of our network is dedicated to performing binary mask segmentation, which serves the purpose of detecting the mask region in the masked face. This initial stage plays a pivotal role in guiding the subsequent stage of our network to complete the missing regions effectively. To achieve this, we introduce the M-Seg module, which utilizes a modified 5 × 5 autoencoder network as shown in M-Seg Module in Figure 2, inspired by the U-Net [25] architecture.

The M-Seg module consists of two main paths: the encoder and decoder paths. The encoding path progressively reduces the spatial dimensions of the feature maps using max pooling. In contrast, the decoding path employs up-convolutional blocks to restore the feature maps to their original size. This architecture facilitates the capture of fine details and spatial consistency by establishing skip connections between corresponding layers of the encoding and decoding paths. These skip connections allow the fusion of low-level and high-level feature representations.

**Figure 3:**
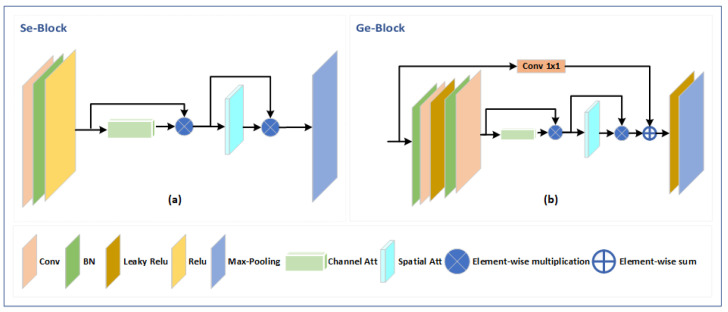
Detailed depiction of the main components: (**a**) Se-Block used for binary mask segmentation in the first stage, and (**b**) Ge-Block representing the generator block.

Within the encoding path of the M-Seg module, each block follows a specific sequence of operations as shown in segmentation encoder (Se)-Block Figure 3a. Firstly, a 3 × 3 convolution is applied to extract spatial features. Batch normalization is then utilized to normalize the output and improve the network’s generalization capability. A rectified linear unit (ReLU) activation function is applied to introduce non-linearity and enhance the network’s ability to capture complex patterns. Additionally, a CBAM block [26] is incorporated to enhance the representation by incorporating channel attention and spatial attention mechanisms. Finally, max-pooling is employed to reduce the spatial dimensions further.

In the decoder path, there are five segmentation decoder (Sd) blocks. The first four blocks consist of up-sampling, a 3 × 3 convolution, batch normalization, and ReLU activation. Up-sampling is used to restore the feature maps to their original size, while the subsequent operations refine the feature representations. The last block in the decoder path utilizes up-sampling, a 1 × 1 convolution, batch normalization, and sigmoid activation. This block is responsible for generating the binary mask map, which indicates the regions of the face covered by the mask. The sigmoid activation function ensures that the output values are 1 for pixels within the mask region and 0 for pixels outside.

The network takes the masked face as input and produces a binary mask map representing the probability of each pixel belonging to the mask region. Leveraging the binary mask segmentation obtained in this first stage, our network can effectively guide the subsequent stage consisting of inpainting the missing regions within the facial images. By accurately delineating the mask regions, our approach ensures reliable completion of the masked regions and generates high-quality reconstructed images.

### 3.2. Face Unmasking Network (FU-Net): GAN-Based Network for Face Unmasking

In this subsection, we present the second stage of our approach, which aims to generate realistic and visually appealing unmasked faces from the input masked faces. Building upon the results of the first stage, where mask regions were detected, this stage focuses on leveraging a generative adversarial network (GAN) to restore the concealed regions while preserving facial details.

#### 3.2.1. GAN Architecture

To accomplish the unmasking task, we propose a GAN-based face unmasking network shown in Figure 2, specifically designed to handle the challenges associated with face mask removal. The generator network adopts an autoencoder architecture inspired by the M-Seg module, tailored to suit the requirements of face unmasking.

The generator network consists of three main components:

(a) Encoder: The encoder incorporates multiple residual attention blocks, denoted as generator encoder (Ge) blocks, which integrate attention mechanisms to enhance the representation of masked faces. These Ge blocks, as depicted in Figure 3b, play a crucial role in capturing important facial features. The encoder is composed of five GE Blocks, with the exception of the last block, where the max-pooling layer is omitted.
(b) Bottleneck: The bottleneck component is a bottleneck layer within the generator network, employing four dilated blocks with dilation rates of 2, 4, 8, and 16, respectively. Each dilated block includes batch normalization (BN), dilated convolutions, and Leaky ReLU activation. This configuration enables effective feature extraction while considering the global context of the masked faces.
(c) Decoder: The decoder follows the architecture of the segmentation network’s decoder path but with Leaky ReLU activation functions instead. It consists of five blocks, with the first lacking an up-sampling layer. This arrangement ensures the reconstruction of unmasked faces with preserved spatial details.

This architecture facilitates the capture of fine details and spatial consistency by establishing skip connections between corresponding layers of the encoding and decoding paths. These skip connections allow the fusion of low-level and high-level feature representations.

In our study, we propose a novel mechanism known as Masked–Unmasked Region Fusion (MURF) to enhance the quality of the generated unmasked faces. The integration of MURF involves combining the original unmasked region with the corresponding region in the generated face through element-wise operations. By performing element-wise multiplications between the masked map and the generated face, as well as between the masked face and the reversed masked map, we obtain intermediate results. These results are then summed together using element-wise summation, leading to improved unmasked faces that effectively utilize information from both the generated and original unmasked regions.

This innovative approach, illustrated in Figure 2, presents a valuable contribution to the field. To ensure the success of MURF, it is crucial to accurately detect the mask region in the first stage, as it significantly influences the overall quality of the final output. By prioritizing precise mask region detection, we can optimize the integration process and achieve superior results in generating realistic unmasked faces.

The discriminator network, on the other hand, comprises a series of convolutional layers followed by leaky ReLU activations. To enhance stability during training, spectral normalization [29] is applied to the discriminator’s convolutional layers. Spectral normalization normalizes the weights of the convolutional filters, ensuring controlled Lipschitz constants and improving the overall network performance.

#### 3.2.2. Perceptual Network

To further enhance the quality and realism of the generated unmasked faces, we incorporate a pre-trained VGG19 network [30] as a perceptual loss [31] component. The VGG network extracts feature representations from the generated and ground truth unmasked faces at multiple layers. By calculating the discrepancy between these feature representations, the perceptual loss guides the training of the generator network, encouraging the generation of visually appealing and realistic unmasked faces that align with the ground truth.

By employing this GAN-based generator, along with the discriminator and the perceptual network, our approach effectively removes face masks while preserving facial details, resulting in high-quality unmasked faces.

### 3.3. Loss Function

#### 3.3.1. M-Seg Loss

In the first stage of our network, we employ a modified autoencoder for binary mask segmentation. To train this stage effectively, we introduce the M-Seg Loss function ($L_{s}$) to measure the discrepancy between the generated binary mask map and the ground truth and quantify their similarity by comparing their overlapping regions. The M-Seg Loss function, as shown in Equation (1), is defined as the combination of two loss functions: binary cross-entropy (*BCE*), represented by Equation (2), and dice loss, which can be defined as stated in Equation (3).

(1) $$L_{s}=BCE+Dice_{loss},$$

(2) $$BCE=-\left(R_{m}\log\left(F_{m}\right)+\left(1-R_{m}\right)\log\left(1-F_{m}\right)\right),$$

(3) $$Dice_{loss}=1-\frac{2\times (|R_{m}\cap F_{m}|)}{|R_{m}\left|+\right|F_{m}|},$$

where $R_{m}$ and $F_{m}$ represent the real and fake masks, respectively.

Binary cross-entropy loss (BCE) is a commonly used loss function in image segmentation tasks. It computes the cross-entropy between the probability distributions of the generated binary mask map and the ground truth, encouraging accurate pixel-wise predictions. We guide the network toward accurately detecting the mask regions by minimizing the binary cross-entropy loss.

Additionally, we incorporate dice loss to further evaluate the similarity between two sets by comparing their overlapping regions. The dice loss is a complementary component to the binary cross-entropy loss in our M-Seg network. By optimizing the joint loss, which combines binary cross-entropy loss and dice loss, our network can improve the pixel-wise predictions and spatial coherence. The joint loss formulation in the first stage plays a critical role in driving the training process and significantly contributes to the overall success of the M-Seg network.

#### 3.3.2. Generator Loss

The generator loss ($L_{joint}$) function in our network is composed of three integral components, adversarial loss for generators ($L_{adv_{g}}$), reconstruction loss ($L_{rec}$), and perceptual loss ($L_{per}$), which work in harmony to guide the training process, as shown in Equation (4).

(4) $$L_{joint}=\alpha L_{adv_{g}}+\beta L_{rec}+\gamma L_{per}$$

where α, β, and γ represent weighted values.

##### Adversarial Loss

Following the principles of least squares generative adversarial networks (LSGANs) [32], our generator aims to produce indistinguishable images from real ones according to the discriminator’s classification. The adversarial loss is computed based on the discriminator’s predictions for the generated images, as presented in Equation (5). By adopting the LSGAN loss, we address the challenges associated with the vanishing gradient problem, which is commonly observed with sigmoid cross-entropy loss. This choice ensures more stable training and facilitates the generation of high-quality and authentic images.

(5) $$L_{adv_{g}}=E_{M_{F}}\left[{\left(D\left(G\left(M_{F}\right)\right)-c\right)}^{2}\right],$$

where $L_{adv_{g}}$ is the generator adversarial loss, $M_{F}$ represents the masked face, and *G* refers to the generator and *D* to the discriminator.

##### Reconstruction Loss

To ensure faithful reconstruction of the input, we incorporate a reconstruction loss composed of two sub-losses, as shown in Equation (6); smoothL1 loss is presented in Equation (7) and SSIM loss ($L_{SSIM}$) is shown in Equation (8). The smoothL1 loss, introduced in Fast R-CNN [33], combines the desirable properties of L1 and L2 losses, balancing robustness and smoothness. By employing this loss, our generator can generate images that exhibit sharp edges and fine details while avoiding excessive noise. Additionally, we integrate the SSIM loss [34], which comprehensively evaluates the structural similarity between the generated and ground truth images, accounting for local and global image characteristics. The reconstruction loss fosters the generation of images that closely resemble the original input, both in terms of pixel-level accuracy and structural coherence.

(6) $$L_{rec}=\frac{1}{2}\left(smoothL1\left(F_{f},R_{f}\right)+L_{SSIM}\right)$$

(7) $$L_{SSIM}=1-SSIM\left(F_{f},R_{f}\right))$$

(8) $$smoothL1\left(F_{f},R_{f}\right)=\left\{\begin{array}{cc} 0.5{\left(R_{f}-F_{f}\right)}^{2}, & \mathrm{if}\;|R_{f}-F_{f}|\;<\;1\\ |R_{f}-F_{f}|-0.5, & \mathrm{otherwise} \end{array}\right.$$

where $R_{f}$ and $F_{f}$ represent real unmasked face and fake unmasked face, respectively.

##### Perceptual Loss

Capturing higher level perceptual characteristics is crucial for generating visually appealing outputs. To achieve this, we incorporate a perceptual loss component. Using a pre-trained VGG19 network [30] as the perceptual network, we extract high-level features from the generated and ground truth images. The perceptual loss ($L_{per}$) is computed by measuring the discrepancy between these feature representations as presented in Equation (9), employing the smoothL1 loss function. This integration allows the generator to learn not only pixel-level similarity but also the intricate perceptual details, resulting in visually appealing images that align with the perceptual qualities of the ground truth.

(9) $$L_{per}=\sum_{i=1}^{n}smoothL1\left(F_{f},R_{f}\right)$$

The generator loss is determined by aggregating these three components using appropriate weighting factors. This joint loss formulation guides the training process, enabling the generator to produce high-quality, realistic, and contextually coherent images, effectively addressing the task of face unmasking.

#### 3.3.3. Discriminator Loss

To compute the discriminator loss function ($L_{adv_{d}}$) and accurately classify real and fake samples, we follow the methodology of least squares generative adversarial networks (LSGANs). The goal is to train the discriminator to output values close to zero for fake samples and values close to one for real samples. We do this by applying the L2 loss function, which compares the predictions of our discriminator with the corresponding target values. It is calculated using Equation (10):

(10) $$L_{adv_{d}}=\frac{1}{2}\left(E_{R_{f}}\left[{\left(D\left(R_{f}\right)-b\right)}^{2}\right]+E_{M_{F}}\left[{\left(D\left(G\left(M_{F}\right)\right)-a\right)}^{2}\right]\right)$$

In Equation (10), we compute the L2 loss between predictions for fake samples and the target value ‘*a*’, as well as between the predictions for real samples and the target value ‘*b*’. To ensure a balanced contribution from fake and real samples, we take the average of these two losses and multiply the sum by 0.5. This formulation provides a stable training signal for adversarial learning, encouraging the discriminator to align its predictions with the desired targets.

In our implementation, we utilize the a-b coding scheme for the discriminator, where ‘*a*’ and ‘*b*’ represent the labels assigned to fake and real data, respectively. This coding scheme facilitates effective discrimination between real and generated samples, enhancing the discriminator’s ability to distinguish between them during adversarial training.

## 4. Experiment and Results

In this section, we present our approach’s experimental setup and results. We begin by describing the dataset employed for training and evaluation purposes. Subsequently, we outline the training procedure, including the specific details and configurations utilized. To assess the performance of our network, we define and utilize appropriate evaluation metrics. Then, we present and discuss our approach’s quantitative and qualitative results, comparing them with state-of-the-art image inpainting methods. Lastly, we conduct a comprehensive analysis of the strengths and limitations exhibited by our approach.

### 4.1. Dataset

**Figure 4:**
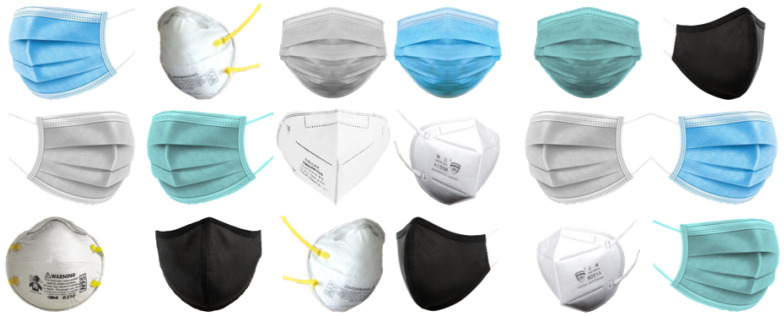
Examples of the diverse masks used in the synthetic dataset. The masks exhibit different shapes, sizes, and colors, comprehensively representing various masking scenarios.

**Figure 5:**
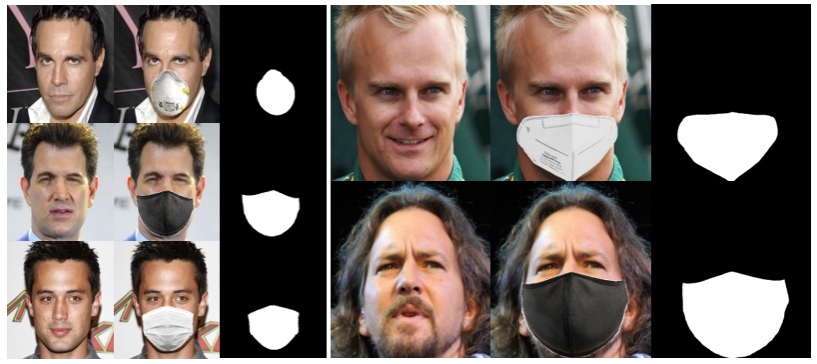
Samples from the synthetic dataset illustrating different sizes (256 × 256 and 512 × 512). The first column displays the original unmasked face images, the second column shows the corresponding masked face images, and the third column represents the binary mask map.

To overcome the scarcity of available datasets containing pairs of masked and unmasked faces, we devised a strategy for generating a synthetic dataset utilizing the publicly accessible CelebA dataset [27]. The CelebA dataset is widely utilized in the computer vision domain, particularly for tasks related to face analysis. It comprises a vast collection of over 200K celebrity images, each annotated with attributes such as gender, age, and facial landmarks. Our approach involved two primary steps in generating the dataset. We initially utilized the facial landmarks provided in the CelebA dataset and the original images. By employing the HD CelebA Cropper [35], we generated two dataset variations containing faces of 256 and 512 pixels. This step ensured the availability of diverse face sizes within our dataset. Subsequently, we employed MaskTheFace [28] to create pairs of masked and unmasked images. MaskTheFace introduced various types of masks with different shapes, colors, and structures into our synthetic dataset. These masks were applied to the 256 and 512 pixel face images, resulting in a dataset containing a diverse range of masked and unmasked faces. Our synthetic dataset consisted of 25K image pairs, each comprising a masked face and its corresponding unmasked version. Additionally, we generated an additional 5K pairs for the purpose of fair comparisons with other state-of-the-art models. We saved the corresponding binary maps for each utilized mask to facilitate further analysis and evaluation, allowing for precise assessment of the generated binary mask maps. For a visual representation of the masks utilized in our dataset, refer to Figure 4. Furthermore, Figure 5 showcases samples from our simulated dataset, providing insights into the diversity and quality of the generated masked and unmasked face pairs. Please note that utilizing the CelebA dataset and the subsequent generation of the synthetic dataset enabled us to address the scarcity of available paired masked and unmasked face datasets, providing a valuable resource for training and evaluating our approach.

### 4.2. Training Procedure Details

We divided the training process into two separate stages during the training phase. We focused on mask map segmentation in the first stage using the M-seg network. The input to the M-seg network was the masked face (MF), and the objective was to generate a binary mask map (MM). This binary mask map served as the second stage’s fourth channel in the input. To evaluate the performance of the first stage, we compared the generated mask map with the corresponding saved binary mask map (ground truth) and updated the network’s weights based on the M-Seg loss.

Moving to the second stage, we trained the FU-Net using the masked face (MF) and the binary mask map (MM) as a four-channel input. The objective of this stage was to generate an unmasked face. We trained the second stage for 100 epochs, while the first stage was trained for 25 epochs. Training was performed on a synthetic dataset comprising 25K image pairs. To ensure proper evaluation, we split the dataset into 80% for training and 20% for validation. Additionally, we utilized an additional 5K testing samples to evaluate the performance of the whole two-stage network. During the training phase, we utilized the saved binary maps for masks that were generated during the dataset generation process.

For the implementation, we utilized PyTorch [36] as the framework for our model. The training was conducted with a batch size of 32 for an image resolution of 256 and a batch size of 8 for an image resolution of 512. We employed a variable learning rate strategy, starting at 0.001 and decreasing it by a factor of 0.1 at specific steps (30,000, 40,000, and 45,000). The training was conducted on an NVIDIA GeForce RTX 4090 GPU with CUDA v12.1, and the operating system used was Windows 11. Both image resolutions, 256 and 512, were trained using this setup.

By following this training procedure and utilizing the specified hardware and software setup, we ensured the effective and efficient training of our model for face unmasking.

### 4.3. Evaluation Metrics

In the evaluation metrics subsection, we will introduce the evaluation metrics used to assess the performance of our model. We will begin by discussing two different metrics employed to evaluate the first stage of the binary mask segmentation task: pixel accuracy and dice score. Subsequently, we will explain the evaluation metrics utilized to assess the performance of our unmasking model and the quality of the generated unmasked faces. These metrics are Peak Signal-to-Noise Ratio (PSNR), Structural Similarity Index Measure (SSIM) [34], Frechet Inception Distance (FID) [37], Naturalness Image Quality Evaluator (NIQE) [38], and Blind/Referenceless Image Spatial Quality Evaluator (BRISQUE) [39], which are widely used in inpainting tasks.

#### 4.3.1. Pixel Accuracy

Pixel accuracy is a commonly used metric for binary segmentation tasks. It measures the percentage of correctly classified pixels in the segmentation mask. By comparing the predicted mask to the ground truth mask, each pixel is classified as correctly classified or misclassified. Pixel accuracy is calculated by the ratio of correctly classified pixels to the total number of pixels in the image, as in Equation (11). While providing an overall measure of the segmentation performance, pixel accuracy does not consider partial overlap or class imbalance.

(11) Pixel Accuracy=correct pixels/Total number of pixels

#### 4.3.2. Dice Score

Dice score, also known as the F1 score, is another widely used metric for binary segmentation tasks. It quantifies the similarity between the predicted and ground truth masks by calculating the overlap between them. The dice score ranges from 0 to 1, with 1 indicating a perfect match between the predicted and ground truth masks. It is computed as the ratio of twice the intersection of the predicted and ground truth masks to the sum of their pixel counts, as shown in Equation (12). The dice score is particularly useful for imbalanced datasets or considering the partial overlap between masks.

(12) $$DiceScore=\frac{2\times (|R_{m}\cap F_{m}|)}{|R_{m}\left|+\right|F_{m}|}$$

These evaluation metrics provide quantitative measures to assess the accuracy and similarity of predicted segmentation masks in the M-seg module. By incorporating both pixel accuracy and dice score, we gain insights into the overall performance of the model, its ability to capture fine details, and its handling of class imbalance.

Moving on to evaluating the quality of the unmasked faces, we utilize PSNR and SSIM metrics.

#### 4.3.3. PSNR

The PSNR is a widely used metric to evaluate the quality of image reconstruction or restoration tasks, including unmasking. It calculates the level of noise or distortion in the generated unmasked face compared to the original unmasked face. It is expressed in decibels (dB) and represents the ratio between the maximum possible pixel value and the mean squared error (MSE) between the original and reconstructed images. A high PSNR indicates low noise or distortion and better image quality. However, the PSNR primarily focuses on pixel-level differences and may not capture perceptual quality or structural information. Equation (13) shows the formula of the PSNR.

(13) $$PSNR=10\log_{10}\left(\frac{R^{2}}{MSE}\right),$$

*R* represents the maximum possible pixel value.
MSE represents the mean squared error between the original and generated faces.

#### 4.3.4. SSIM

The SSIM is a perceptual metric that assesses the similarity between the original and generated images. It considers not only pixel-level differences but also structural information such as texture, edges, and contrast. Three terms are calculated in the SSIM, as shown in Equation (14): luminance similarity, contrast similarity, and structural similarity. Combining these terms provides an overall index ranging from −1 to 1, where 1 indicates a perfect match between the images. The SSIM is preferred over the PSNR for evaluating image quality as it considers perceptual aspects, making it more suitable for assessing unmasking models.

(14) $$SSIM\left(R_{f},F_{f}\right)=\frac{\left(2\mu_{R_{f}}\mu_{F_{f}}+C_{1}\right)+\left(2\sigma_{R_{f}F_{f}}+C_{2}\right)}{\left(\mu_{R_{f}}^{2}+\mu_{F_{f}}^{2}+C_{1}\right)\left(\sigma_{R_{f}}^{2}+\sigma_{F_{f}}^{2}+C_{2}\right)}$$

$\mu_{R_{f}}$ represents the average of the original face.
$\mu_{F_{f}}$ represents the average of the generated face.
$\sigma_{R_{f}}$ represents the variance of the original face.
$\sigma_{F_{f}}$ represents the variance of the generated face.
$\sigma_{R_{f}F_{f}}$ represents the variance of the original and generated face.
$C_{1}$, $C_{2}$ are two variables to stabilize the division operation. We use $C_{1}={\left(0.01\right)}^{2}$ and $C_{2}={\left(0.03\right)}^{2}$.

#### 4.3.5. FID

The FID measures the similarity between the distribution of generated images and the distribution of real images in a high-dimensional feature space extracted from a pre-trained InceptionV3 network. A lower FID score indicates a higher similarity between the distributions and, therefore, better image quality. Equation (15) shows the FID formula.

(15) $$FID\left(R_{f},F_{f}\right)=\left|\right|\mu_{R_{f}}-\mu_{F_{f}}{\left|\right|}^{2}+Tr\left(C_{R_{f}}+C_{F_{f}}-2{\left(C_{R_{f}}-C_{F_{f}}\right)}^{\frac{1}{2}}\right)$$

$\mu_{R_{f}}$ represents the mean of the features extracted from the original face.
$\mu_{F_{f}}$ represents the mean of the features extracted from the generated face.
$C_{R_{f}}$ represents the covariance matrix of the original feature vector.
$C_{F_{f}}$ represents the covariance matrix of the generated feature vector.
Tr() denotes the trace of a matrix, which is the sum of the diagonal elements.

#### 4.3.6. NIQE

NIQE is a no-reference metric that assesses the naturalness and perceptual quality of images. It quantifies the level of distortion or artifacts present in the generated images without requiring a reference image. A lower NIQE score suggests the generated images have higher naturalness and visual quality.

#### 4.3.7. BRISQUE

BRISQUE is a no-reference metric that evaluates the spatial quality of images. It measures the amount of artifacts and distortions present in the images, considering structural and textural information. A lower BRISQUE score indicates better image quality in terms of spatial information.

We can evaluate different aspects of our unmasking model’s performance by utilizing PSNR, SSIM, FID, NIQE, and BRISQUE metrics. The PSNR focuses on pixel-level differences, while the SSIM captures perceptual similarity and structural information, the FID evaluates the distribution similarity between generated and real images, NIQE assesses naturalness, and BRISQUE measures spatial quality. These metrics provide quantitative measures to assess the quality and similarity of the unmasked images compared to the original ones, enabling us to evaluate the effectiveness of our model in preserving image details and reducing distortion.

### 4.4. Results

In this subsection, we evaluate the performance of our GANMasker model for realistic face mask removal and compare it with state-of-the-art methods. We provide a comprehensive analysis that combines qualitative and quantitative assessments, showcasing the effectiveness and advantages of our approach.

#### 4.4.1. First Stage

We conducted an evaluation to assess the effectiveness of the first stage of our approach, which focuses on mask map segmentation. The first stage achieved exceptional results, with pixel accuracy reaching 99.99% and the dice metric achieving 99.97%. These impressive scores highlight the accuracy and precision of the first stage, which significantly influence the overall performance of our approach.

#### 4.4.2. Second Stage

##### Qualitative Comparison

**Figure 6:**
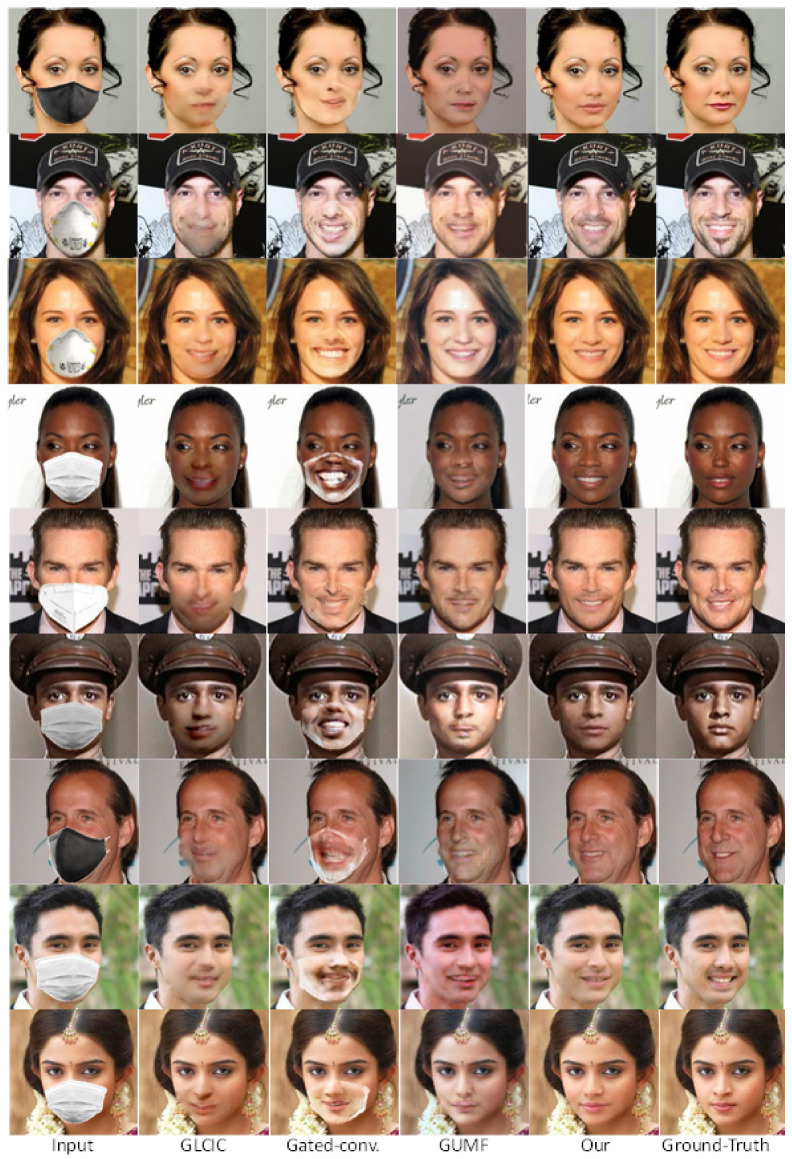
Qualitative comparison with state-of-the-art methods. The first column shows the input masked images, followed by four output columns obtained by GLCIC, Gated-Conv, GUMF, and our model, respectively, while the last column displays the corresponding ground truth images.

**Figure 7:**
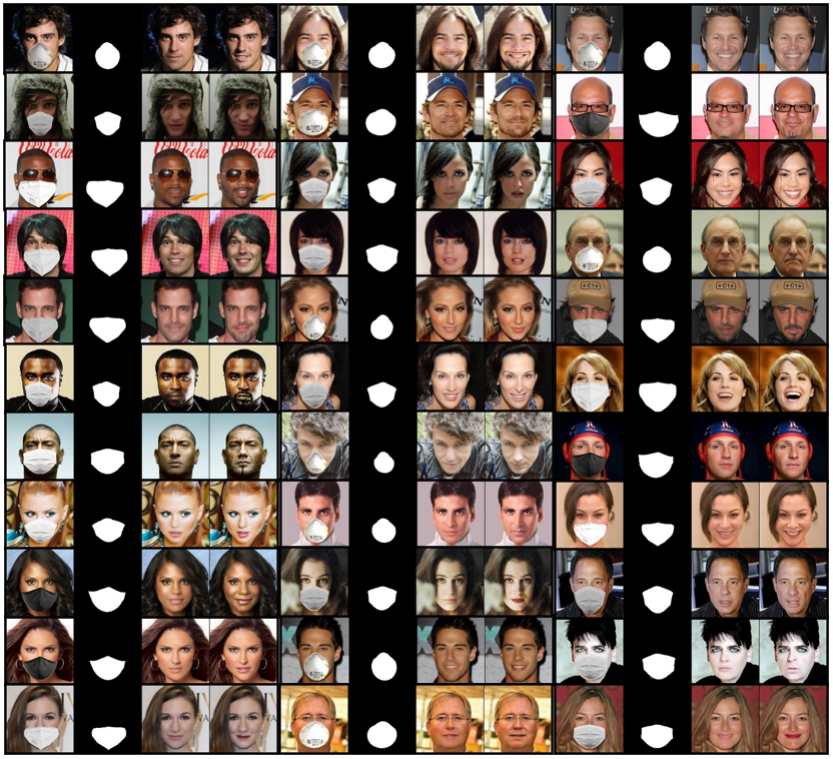
Some samples of the proposed model’s results on front-facing images. Each sample includes the input masked face, the generated binary mask map, the proposed model’s output, and the ground truth.

**Figure 8:**
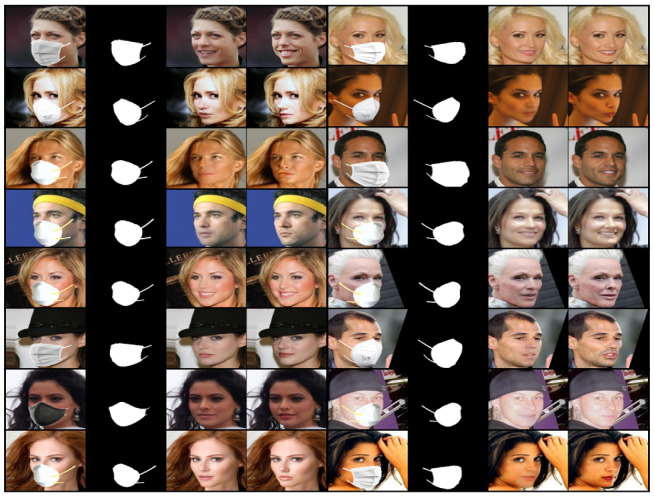
Some samples of the proposed model’s results on side-facing images. Each sample includes the input masked face, the generated binary mask map, the proposed model’s output, and the ground truth.

**Figure 9:**
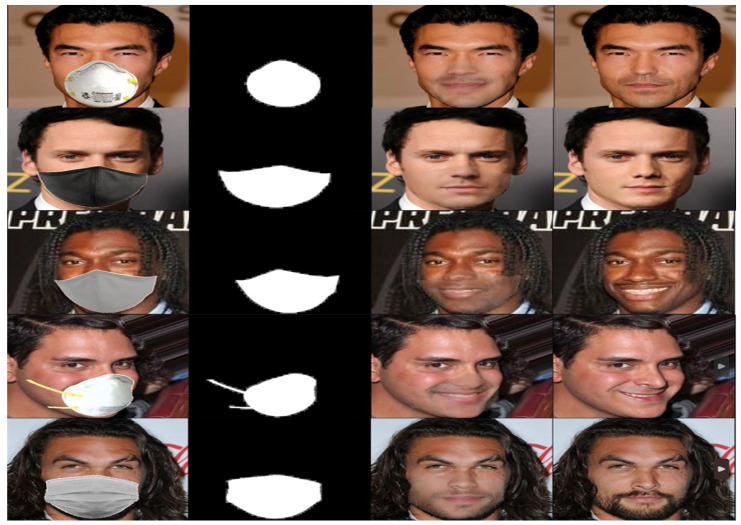
Sample results of our model on images of size 512 × 512, demonstrating its effectiveness on various mask types, sizes, angles, and colors. The first column depicts the masked input face, followed by the binary mask map generated in the first stage. The third column showcases our model’s output, while the fourth column presents the corresponding ground truth for comparison.

We visually compared the performance of our GANMasker model with three prominent state-of-the-art methods for face mask removal: GLCIC [12], GUMF [18], and Gated Conv [13]. Figure 6 showcases the generated unmasked faces using our model and the other approaches. The comparison clearly illustrates that our model produces the best results among all the methods, with outputs that are more realistic, natural, and closer to the ground truth. By presenting side-by-side comparisons, we highlight the visual quality, detail preservation, and realism achieved by our model. Moreover, Figure 7 and Figure 8 demonstrate the efficacy and versatility of the proposed model’s performance. Figure 7 showcases the results on frontal faces, while Figure 8 presents the results on side faces. These two figures, in addition to Figure 6, collectively illustrate the diversity in mask types, colors, and sizes, as well as the wide range of facial attributes, including gender, age, and skin color, that our model adeptly handles. Figure 9 presents our model’s results on 512 × 512 images, further demonstrating the effectiveness of our approach on high-resolution images. These qualitative comparisons emphasize the superior performance of our approach, showcasing visually convincing and artifact-free unmasked faces.

##### Quantitative Comparison

To ensure an objective evaluation, we quantitatively compared our GANMasker model with three state-of-the-art methods: GLCIC [12], GUMF [18], and Gated Conv [13]. For a comprehensive assessment, we employed five evaluation metrics: Peak Signal-to-Noise Ratio (PSNR), Structural Similarity Index (SSIM), Frechet Inception Distance (FID), Naturalness Image Quality Evaluator (NIQE), and Blind/Referenceless Image Spatial Quality Evaluator (BRISQUE). The PSNR, SSIM, and FID are widely used as referenced metrics, while NIQE and BRISQUE are non-referenced metrics that offer valuable insights into image quality. Despite the challenges in quantitative evaluation metrics for image inpainting, we leverage these well-established metrics to evaluate the quality, naturalness, fidelity, and perceptual similarity of the unmasked images generated by models compared to the ground truth. The evaluation results, including the PSNR, the SSIM, the FID, NIQE, and BRISQUE, on the validation images of the CelebA dataset are presented in Table 1. Our GANMasker model achieves higher scores in the PSNR, the eSSIM, the FID, and NIQE than the existing methods, indicating superior image fidelity and similarity to the ground truth. Including these diverse metrics strengthens our confidence in the effectiveness of GANMasker and its outperformance against state-of-the-art methods. The IQA-PyTorch toolbox [40] was employed to compute the FID, NIQE, and BRISQUE scores for all the models.

This comprehensive evaluation establishes the effectiveness and superiority of our proposed GANMasker approach for realistic face mask removal. The remarkable first stage results in mask map segmentation lay the foundation for the overall success of our approach. The qualitative comparison with GLCIC [12], GUMF [18], and Gated Conv [13] visually confirms the superior performance of our model. The quantitative comparison utilizing the PSNR, the SSIM, the FID, NIQE, and BRISQUE provides objective measures supporting our claims of higher quality and fidelity. These results, along with Figure 6, Figure 7, Figure 8 and Figure 9, highlight the significant advancements offered by GANMasker in the field of face mask removal.

#### 4.4.3. Exploring Limitations: Analysis of Challenging Cases and Failed Results

**Figure 10:**
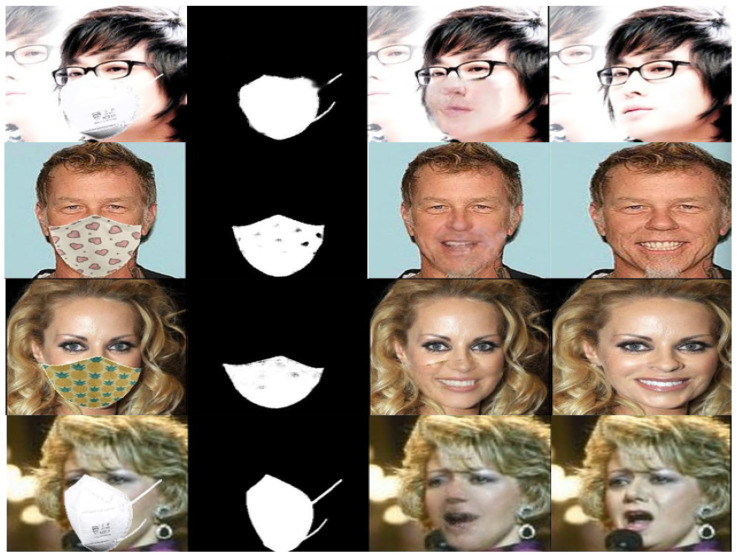
Failure results—challenging cases and mask map detection errors.

While our approach demonstrates strong performance in unmasking images, examining its limitations and understanding its challenges in certain scenarios is crucial. In this subsection, we comprehensively analyze challenging cases where our model encountered difficulties in accurately recovering the underlying content from heavily occluded or complex masked images. Additionally, we address situations where the first stage fails to detect the mask map correctly, particularly for large or differently colored masks that were not adequately represented during the training phase. By carefully examining these failure results, we aim to gain valuable insights into the specific limitations and areas for improvement of our approach. Figure 10 showcases these failure results, shedding light on our challenges and providing valuable insights for future research and enhancements.

### 4.5. Ablation Study: Evaluating the Impact of the First Stage

In this subsection, we delve into an ablation study to assess the significance of the first stage, specifically the mask segmentation module, on the overall performance of our model. We aim to evaluate how incorporating the mask segmentation module thoroughly enhances the results of face unmasking.

To ensure a fair comparison, we meticulously compared two variants of our model: a two-stage model including both stages and a one-stage model excluding the first stage. By conducting this study, we can gain valuable insights into the specific contributions and benefits of the mask segmentation module.

Our experimental setup remained consistent throughout the study, employing the same dataset, hyperparameters, and evaluation metrics for both models. This allows us to isolate and accurately measure the impact of the first stage on the overall performance of our approach.

The results of our ablation study are compelling. The two-stage model consistently outperforms the one-stage model in terms of both quantitative and qualitative assessments. We present the quantitative metrics, including the SSIM and the PSNR, in Table 2, highlighting the superior performance of the two-stage model. These metrics indicate higher image fidelity and a closer similarity to the ground truth, validating the effectiveness of the mask segmentation module.

**Figure 11:**
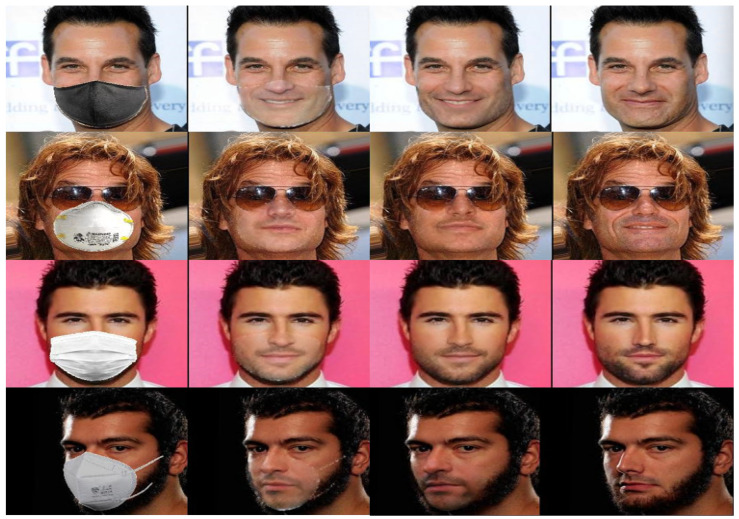
Comparison of face unmasking results between the two- and one-stage models. The left column shows input masked faces, followed by the outputs from the one-stage model. The third column displays outputs from the two-stage model, while the last column presents ground truth unmasked faces.

Furthermore, we showcase visual examples of the final results of the one-stage and two-stage models in Figure 11. Through these qualitative comparisons, we demonstrate the significant improvements achieved by the two-stage model. The unmasked faces generated by this model exhibit enhanced realism, naturalness, and visual appeal.

The findings of our ablation study strongly confirm the mask segmentation module’s substantial contribution to enhancing our approach’s overall performance. The superiority of the two-stage model over the one-stage model provides evidence for the effectiveness of the first stage in achieving superior face unmasking results.

## 5. Conclusions and Future Work

In this study, we introduced GANMasker, a powerful two-stage GAN-based approach for realistic face mask removal. Our evaluations and comparisons with state-of-the-art methods clearly demonstrated the effectiveness and superiority of our approach. GANMasker effectively recovers fine details and accurately reconstructs underlying information of masked images by leveraging advanced deep learning techniques and incorporating attention mechanisms, contextual information, and feature extraction modules. We achieved visually convincing and artifact-free unmasked faces thanks to the remarkable results obtained in the first stage, which focused on mask map segmentation.

Moving forward, there are exciting directions for future research and improvements in face mask removal, such as enhancing the performance of our model in challenging scenarios, including severe occlusion or complex mask patterns. Additionally, we see great potential in integrating our technique with facial recognition research. By removing masks as a preprocessing step, we can improve the accuracy and reliability of facial recognition systems.

To summarize, GANMasker represents a significant breakthrough in realistic face mask removal. Our approach has demonstrated superior performance, highlighted promising research directions, and opened up possibilities for enhancing the accuracy of facial recognition systems. We are excited about the impact of GANMasker and look forward to further advancements in this field.

## Figures and Tables

**Table 1:** Quantitative comparison of our method with different approaches in terms of Structural Similarity (SSIM), Peak Signal-to-Noise Ratio (PSNR), Frechet Inception Distance (FID), Naturalness Image Quality Evaluator (NIQE), and Blind/Referenceless Image Spatial Quality Evaluator (BRISQUE). The bold results represent the best performance in each column.

Method	SSIM	PSNR	FID	NIQE	BRISQUE
GLCIC [12]	0.94	26.78	16.52	5.99	18.48
GatedConv [13]	0.89	20.57	90.81	5.75	**17.63**
GUMF [18]	0.90	21.69	58.71	6.45	32.93
Ours	**0.95**	**30.96**	**16.34**	**5.42**	19.27

**Table 2:** Quantitative comparison of our method with different approaches in terms of Structural Similarity (SSIM) and the Peak Signal-to-Noise Ratio (PSNR). The bold results represent the best performance in each column.

Method	SSIM	PSNR
Our (one-stage)	0.94	28.18
Our (two-stage)	**0.95**	**30.96**

## Data Availability

The datasets used in this paper are public datasets.

## References

[1] Jassim F.A. (2013). Image inpainting by Kriging interpolation technique. arXiv.

[2] Alsalamah M., Amin S. (2016). Medical image inpainting with RBF interpolation technique. Int. J. Adv. Comput. Sci. Appl..

[3] Criminisi A., Pérez P., Toyama K. (2004). Region filling and object removal by exemplar-based image inpainting. IEEE Trans. Image Process..

[4] Barnes C., Shechtman E., Finkelstein A., Goldman D.B. (2009). PatchMatch: A randomized correspondence algorithm for structural image editing. ACM Trans. Graph..

[5] Liu J., Musialski P., Wonka P., Ye J. (2012). Tensor completion for estimating missing values in visual data. IEEE Trans. Pattern Anal. Mach. Intell..

[6] Simakov D., Caspi Y., Shechtman E., Irani M. Summarizing visual data using bidirectional similarity. Proceedings of the 2008 IEEE Conference on Computer Vision and Pattern Recognition.

[7] Darabi S., Shechtman E., Barnes C., Goldman D.B., Sen P. (2012). Image melding: Combining inconsistent images using patch-based synthesis. ACM Trans. Graph. (TOG).

[8] Bertalmio M., Sapiro G., Caselles V., Ballester C. Image inpainting. Proceedings of the 27th Annual Conference on Computer Graphics and Interactive Techniques.

[9] Prasath V.S., Thanh D.N., Hai N.H., Cuong N.X. Image restoration with total variation and iterative regularization parameter estimation. Proceedings of the 8th International Symposium on Information and Communication Technology.

[10] Biradar R.L., Kohir V.V. (2013). A novel image inpainting technique based on median diffusion. Sadhana.

[11] Pathak D., Krahenbuhl P., Donahue J., Darrell T., Efros A.A. Context encoders: Feature learning by inpainting. Proceedings of the IEEE Conference on Computer Vision and Pattern Recognition.

[12] Iizuka S., Simo-Serra E., Ishikawa H. (2017). Globally and locally consistent image completion. ACM Trans. Graph. (ToG).

[13] Yu J., Lin Z., Yang J., Shen X., Lu X., Huang T.S. Free-form image inpainting with gated convolution. Proceedings of the IEEE/CVF International Conference on Computer Vision.

[14] Nazeri K., Ng E., Joseph T., Qureshi F.Z., Ebrahimi M. (2019). Edgeconnect: Generative image inpainting with adversarial edge learning. arXiv.

[15] Liu G., Reda F.A., Shih K.J., Wang T.C., Tao A., Catanzaro B. Image inpainting for irregular holes using partial convolutions. Proceedings of the European Conference on Computer Vision (ECCV).

[16] Li Y., Liu S., Yang J., Yang M.H. Generative face completion. Proceedings of the IEEE Conference on Computer Vision and Pattern Recognition.

[17] Khan M.K.J., Ud Din N., Bae S., Yi J. (2019). Interactive removal of microphone object in facial images. Electronics.

[18] Din N.U., Javed K., Bae S., Yi J. (2020). A novel GAN-based network for unmasking of masked face. IEEE Access.

[19] Jam J., Kendrick C., Drouard V., Walker K., Hsu G.S., Yap M.H. R-mnet: A perceptual adversarial network for image inpainting. Proceedings of the IEEE/CVF Winter Conference on Applications of Computer Vision.

[20] Kasem M., Abdallah A., Berendeyev A., Elkady E., Abdalla M., Mahmoud M., Hamada M., Nurseitov D., Taj-Eddin I. (2022). Deep learning for table detection and structure recognition: A survey. arXiv.

[21] Abdallah A., Berendeyev A., Nuradin I., Nurseitov D. (2022). Tncr: Table net detection and classification dataset. Neurocomputing.

[22] Ibrahem H., Salem A., Kang H.S. (2022). Exploration of Semantic Label Decomposition and Dataset Size in Semantic Indoor Scenes Synthesis via Optimized Residual Generative Adversarial Networks. Sensors.

[23] Hamada M.A., Abdallah A., Kasem M., Abokhalil M. Neural network estimation model to optimize timing and schedule of software projects. Proceedings of the 2021 IEEE International Conference on Smart Information Systems and Technologies (SIST).

[24] Mahmoud M., Kasem M., Abdallah A., Kang H.S. AE-LSTM: Autoencoder with LSTM-Based Intrusion Detection in IoT. Proceedings of the 2022 International Telecommunications Conference (ITC-Egypt).

[25] Ronneberger O., Fischer P., Brox T. (2015). U-net: Convolutional networks for biomedical image segmentation. Proceedings of the Medical Image Computing and Computer-Assisted Intervention–MICCAI 2015: 18th International Conference, Munich, Germany, 5–9 October 2015, Proceedings, Part III 18.

[26] Woo S., Park J., Lee J.Y., Kweon I.S. Cbam: Convolutional block attention module. Proceedings of the European Conference on Computer Vision (ECCV).

[27] Liu Z., Luo P., Wang X., Tang X. (2018). Large-scale celebfaces attributes (celeba) dataset. Retrieved August.

[28] Anwar A., Raychowdhury A. (2020). Masked face recognition for secure authentication. arXiv.

[29] Miyato T., Kataoka T., Koyama M., Yoshida Y. (2018). Spectral normalization for generative adversarial networks. arXiv.

[30] Simonyan K., Zisserman A. (2014). Very deep convolutional networks for large-scale image recognition. arXiv.

[31] Johnson J., Alahi A., Fei-Fei L. (2016). Perceptual losses for real-time style transfer and super-resolution. Proceedings of the Computer Vision–ECCV 2016: 14th European Conference, Amsterdam, The Netherlands, 11–14 October 2016, Proceedings, Part II 14.

[32] Mao X., Li Q., Xie H., Lau R.Y., Wang Z., Paul Smolley S. Least squares generative adversarial networks. Proceedings of the IEEE International Conference on Computer Vision.

[33] Girshick R. Fast r-cnn. Proceedings of the IEEE International Conference on Computer Vision.

[34] Wang Z., Bovik A.C., Sheikh H.R., Simoncelli E.P. (2004). Image quality assessment: From error visibility to structural similarity. IEEE Trans. Image Process..

[35] LynnHo (2019). HD-CelebA-Cropper. https://github.com/LynnHo/HD-CelebA-Cropper.git.

[36] Paszke A., Gross S., Massa F., Lerer A., Bradbury J., Chanan G., Killeen T., Lin Z., Gimelshein N., Antiga L. (2019). Pytorch: An imperative style, high-performance deep learning library. Adv. Neural Inf. Process. Syst..

[37] Heusel M., Ramsauer H., Unterthiner T., Nessler B., Hochreiter S. (2017). Gans trained by a two time-scale update rule converge to a local nash equilibrium. Adv. Neural Inf. Process. Syst..

[38] Mittal A., Soundararajan R., Bovik A.C. (2012). Making a “completely blind” image quality analyzer. IEEE Signal Process. Lett..

[39] Mittal A., Moorthy A.K., Bovik A.C. (2012). No-reference image quality assessment in the spatial domain. IEEE Trans. Image Process..

[40] Chen C., Mo J. (2022). IQA-PyTorch: PyTorch Toolbox for Image Quality Assessment. https://github.com/chaofengc/IQA-PyTorch.

